# Laboratory preparation methods for human-derived fecal microbial suspensions for fecal microbiota transplantation: a review and standardization perspectives

**DOI:** 10.3389/fmicb.2025.1637673

**Published:** 2025-09-03

**Authors:** Jinhua Gong, Yuchi Liu, Liuye Huang

**Affiliations:** ^1^Department of Gastroenterology, The Affiliated Yantai Yuhuangding Hospital of Qingdao University, Yantai, China; ^2^Qingdao Medical College, Qingdao University, Qingdao, China

**Keywords:** fecal microbiota transplantation, fecal suspension preparation, laboratory preparation, protocol standardization, microbial viability, gut microbiota diversity

## Abstract

Fecal microbiota transplantation (FMT) has advanced significantly as a therapeutic approach over the past few decades. Preparing fecal suspensions for FMT is one of the key steps. However, there is no unified standard or recognized procedure for preparing fecal suspensions in laboratories. This review evaluated the steps currently employed in laboratories to prepare fecal suspensions for FMT, including sample collection, suspension buffers, homogenization, purification, filtration, centrifugation, cryopreservation, dosage, the operating environment, and the transplantation form. This review focuses on the different operations of each preparation step, aiming to provide a reference for the laboratory preparation of fecal suspensions.

## 1 Introduction

The human gut microbiota, often referred to as the “second genome,” constitutes a dynamic ecosystem of trillions of microbial cells that encode more than 3 × 10^6^ genes (Qin, 2010). Dysbiosis of this ecosystem has been implicated in a wide range of diseases, ranging from gastrointestinal disorders such as inflammatory bowel disease and *Clostridioides difficile* infection to systemic conditions such as diabetes, neurodegenerative diseases, and metabolic syndrome (El Hage Chehade, 2023; Sokol, 2020; van Nood et al., 2013; Zhang et al., 2024; Jensen et al., 2022; Góralczyk-Bińkowska et al., 2022). Fecal microbiota transplantation (FMT), a therapeutic intervention involving the transfer of processed fecal material from a healthy donor to a recipient, has emerged as a powerful tool to restore gut microbial homeostasis (Shi and Zhang, 2020; Cammarota et al., 2017).

However, in current studies, FMT efficacy varies in the same disease (Karimi et al., 2024). These discrepancies may arise from racial and dietary variations but could also stem from the heterogeneous methods used to prepare the fecal bacterial suspensions. The success of FMT depends heavily on the effective preparation of fecal microbiota suspensions, which must preserve the viability and diversity of the microbial community to ensure successful engraftment and therapeutic efficacy (Karimi et al., 2024). Given the critical role of microbial viability and the diversity of the microbial community in FMT, it is essential to optimize the preparation and preservation methods to maximize the therapeutic potential of fecal microbiota suspensions. The preparation methods for fecal microbiota suspensions vary widely, and currently, no standardized procedure has been widely accepted (Secombe et al., 2021).

**Figure 1:**
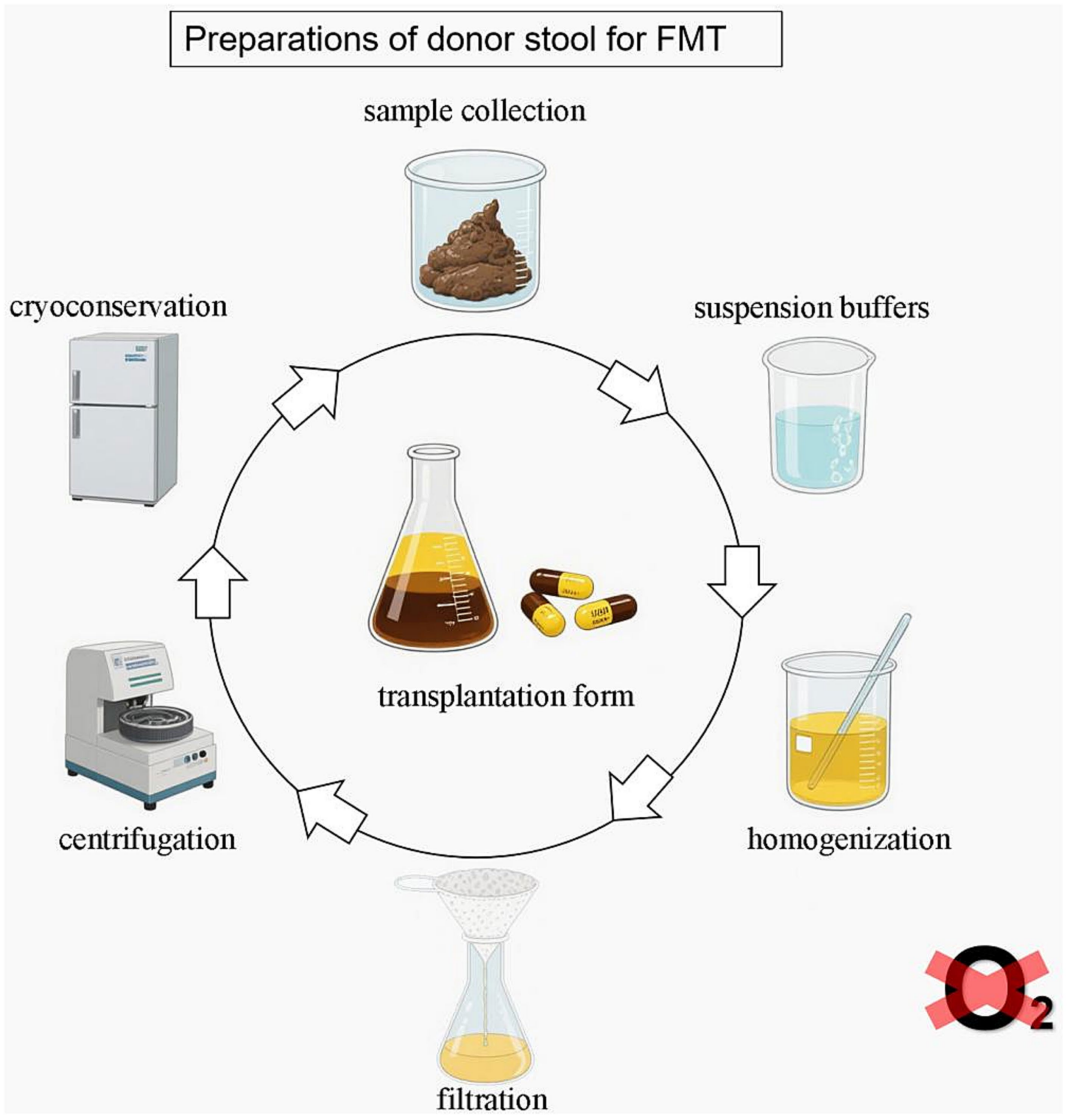
Preparation of donors for FMT: a standardized laboratory workflow.

This review aims to summarize the current methods for preparing fecal microbiota suspensions, highlight their impact on microbial viability and diversity, and explore future directions for standardizing these procedures to increase the safety and efficacy of FMT. The current process involved in FMT is shown in Figure 1.

The standardization of fecal suspension preparation is not merely a methodological concern but a prerequisite for regulatory compliance and clinical translation. Standardization of FMT procedures is essential for regulatory approval, particularly in the context of Investigational New Drug (IND) applications. The US Food and Drug Administration (FDA) has emphasized the importance of potency assays as part of the IND application process for FMT products. These assays are crucial for ensuring the efficacy and safety of FMT treatments (Thanush et al., 2023). For instance, the FDA has approved two FMT-based products, Rebyota and Vowst (Deepti et al., 2025), which underwent rigorous testing and validation to meet regulatory standards. Furthermore, the regulatory landscape for FMT varies globally. While FMT is regulated as a drug in the United States, it is classified as a medicinal product in the United Kingdom and as a transplant product in some European countries (Thanush et al., 2023; Deepti et al., 2025; Merrick et al., 2020). This variation underscores the importance of standardization to facilitate international acceptance and application of FMT. In conclusion, standardization of FMT procedures is critical for meeting regulatory requirements, ensuring product safety and efficacy, and facilitating broader clinical application.

Laboratory workflow for FMT fecal suspension preparation. The steps used in laboratories to prepare fecal suspensions for FMT include sample collection, suspension buffers, homogenization, purification (filtration, centrifugation), cryopreservation, dosage, operating environment, and transplantation. The preparation of donor stool is recommended in an anaerobic environment.

## 2 Sample collection

Fecal sample collection is the first step in the laboratory preparation of fecal bacterial suspensions and serves as a fundamental procedure in studies. The sample collection process included two steps: fresh fecal sample collection and postcollection transportation. For the collection of fresh human fecal samples, natural excrement is collected, and sterile disposable sampling tools are predominantly recommended (Chen, 2022; Yu et al., 2023). During collection, fecal samples must be taken care of to avoid contamination of the sample by blood or urine (Perez et al., 2016). In the preparation of rodent-derived fecal bacterial suspensions, most studies use fecal pellets, while some employ gastrointestinal contents as the source material (Secombe et al., 2021). Although 30 g of fecal weight has been demonstrated to be sufficient for successful FMT (Satokari et al., 2015; Costello et al., 2016), current guidelines increasingly advocate for the collection of at least 50 g of human fecal material (Shi and Zhang, 2020; Chen, 2022; Yu et al., 2023), as larger volumes are correlated with higher success rates in subsequent FMT procedures (Gough et al., 2011).

During transportation to the laboratory, samples should be maintained at 4°C with minimal transit duration (Burz et al., 2019). Prolonged storage at room temperature (>24 h) has been shown to increase the abundance of Actinobacteria and decrease that of Firmicutes (Choo et al., 2015). Unlike metagenomic sequencing and other analytical approaches, FMT necessitates the colonization of viable bacteria within the intestinal tract. Fecal samples intended for microbial transplantation should not be directly frozen; otherwise, the formation and growth of ice crystals within cells, membrane rupture, and chemical osmotic stress during freezing can cause damage leading to bacterial death, especially increasing the mortality rate of Bacteroides (Chen et al., 2022). Current evidence indicates no significant decline in microbial viability when fecal samples are stored at 4°C for 6–8 h (h); however, bacterial mortality increases with increasing temperature (Burz et al., 2019; Ott et al., 2004; Cammarota et al., 2019). After 8 h of transportation, the activity and diversity of bacterial groups decreased significantly, and after 24 h, the decline was more obviou (Ott et al., 2004). The European consensus advocates limiting the timeframe between collecting donor feces and transplanting bacterial liquid into recipients or storing it to within 6 h (Cammarota et al., 2017; Cammarota et al., 2019; König et al., 2017). The Nanjing consensus on the methodology of washed microbiota transplantation indicates that, on the basis of the smart isolation and purification system, the “FMT 1 h protocol” can best preserve functional bacterial communities (e.g., intestinal anaerobic symbiotic bacteria) and their ability to synthesize key anti-inflammatory metabolites, likely enhancing the clinical response rate of FMT for inflammatory bowel disease (Shi and Zhang, 2020).

## 3 Suspension buffers

The selection of appropriate suspension buffers determines the microbial viability and suspension quality in FMT. Phosphate-buffered saline (PBS) is often chosen as the suspension buffer. Alternative buffers include filtered autoclaved water (Ericsson, 2017), 200-proof ethanol (Lin et al., 2019) and normal saline. Normal saline has limited buffering capacity and cannot effectively stabilize the pH value. Moreover, lacking any protective components, it provides minimal protection for oxygen-sensitive anaerobic bacteria. The core advantage of PBS lies in its phosphate buffer system, which maintains the solution’s pH at neutral levels (Zhang et al., 2020; Zhang et al., 2023). Maintaining stable neutral pH is crucial for preserving the integrity of cell membranes and key enzyme activities in most intestinal bacteria (Karki et al., 2020; Lindsay, 2015). However, PBS still fails to address oxidative damage issues. Some methods incorporate protective additives (e.g., cysteine hydrochloride or L-cysteine) into PBS (Secombe et al., 2021). L-cysteine (0.05 g/L) plays a role in protecting anaerobic bacteria from oxidative damage, thereby maintaining the integrity of the microbial population (Jingjing, 2024).

The buffer system in the widely adopted intelligent fecal microbiota processing system remains normal saline (Yu et al., 2023). It is recommended that the mixing ratio of the feces be adjusted during FMT preparation (Rasmussen et al., 2019). In terms of buffer volume, fecal-to-buffer ratios are typically maintained between 1: 3 and 1:10 (w/v; Stebegg et al., 2019; Bárcena et al., 2019). According to He *et al*., fresh stool (25%) should be homogenized with normal saline (60%) and pharmaceutical-grade glycerol (15%) via an automatic stirring and separation machine to prepare fecal microbiota transplantation (FMT) material. This standardized protocol ensures the optimal consistency and viability of the microbial community during processing (He et al., 2021).

## 4 Homogenization

Following the addition of appropriate suspension buffers, achieving optimal fecal homogenization is an important step. Unlike homogeneous biofluids such as urine, fecal samples exhibit inherent structural heterogeneity, with demonstrated spatial variability in microbial composition across different fecal regions (Liang et al., 2020; Gorzelak, 2015). Current homogenization methodologies include manual stirring (e.g., using mortars and pestles or Dounce homogenizers), hand agitation, vortex mixing (e.g., Vortex-Genie 2 or Denagene Vortex Mixer), mechanical oscillation (e.g., Next, Advance Bullet Blender or FLUKO Pneumatic Mixers), blender-based processing (e.g., FLUKO Electric Overhead Stirrers or FLUKO Handheld Homogenizers; Shi and Zhang, 2020; Stephen and Cummings, 1980; Hsieh et al., 2016; Lee et al., 2024; Wang et al., 2019; Yadegar et al., 2024), and automatic stirring and separation machines (He et al., 2021). However, there is no methodological consensus on technique selection, which is complicated by insufficient procedural documentation in published studies (Secombe et al., 2021).

## 5 Purification

During fecal microbiota suspension preparation, particularly in laboratory settings, effective impurity removal involves three principal techniques: sedimentation, filtration, and centrifugation. While some protocols employ singular methodologies, others combine filtration with centrifugation (Xiao et al., 2025). For instance, Hamilton *et al*. implemented sequential filtration through 2.0 mm, 1.0 mm, 0.5 mm, and 0.25 mm stainless steel laboratory sieves, followed by microbial enrichment via centrifugation at 6,000 × g for 15 min (min; Hamilton et al., 2012). Notably, automated purification systems have emerged as advantageous alternatives, offering enhanced processing efficiency, standardized protocols, and reduced operator exposure to fecal material (Shi and Zhang, 2020; Zhang et al., 2020). On the one hand, coarse filtration induces minimal alterations to the microbial community structure, whereas centrifugation has the most pronounced impact on both microbial abundance and community architecture (Mi et al., 2023); on the other hand, higher bacterial mortality rates are detected in centrifuged samples than in those processed through sedimentation (Liu et al., 2020).

### 5.1 Filtration

Filtration is a widely used purification strategy. Common filtration tools include sterile gauze, plastic membranes, and metal mesh sieves. Sterile gauze is typically employed for the initial removal of large particulate matter, whereas plastic membranes and metal sieves enable finer filtration.

Filtration methodologies employ pore sizes ranging from nonstandardized gauze to standardized meshes (e.g., 200-, 400-, 800-, or 1,000-mesh), with configurations spanning single-layer to multilayer systems. While sterile gauze is the predominant choice for primary filtration because of its accessibility and cost-effectiveness (Jamshidi et al., 2023; Zhao et al., 2023; Bokoliya et al., 2021), alternative approaches have been explored. For example, Brunse et al. utilized 70 μm cell strainers to minimize particulate interference (Brunse et al., 2022), whereas Hu et al. implemented 0.25 mm stainless steel mesh screens for enhanced particle removal (Hu et al., 2018). Notably, the selection of pore size requires careful consideration of microbial dimensions: human fecal bacteria typically range from 0.89--1.4 μm in diameter, with bacterial aggregates reaching up to 4 μm in diameter (Oinam et al., 2022; Mortimer et al., 2016). Although sequential single-layer filtration with graded pore sizes is widely adopted, multilayer filtration is increasingly recommended because of its operational advantages. This approach reduces the clogging frequency, decreases filter replacement demands, enhances bacterial viability through reduced shear stress, and improves operator safety by minimizing direct exposure to biohazardous materials. Zhang et al. reported that the integration of microfiltration (via an automated purification system) with three iterative centrifugation–resuspension cycles in a washed microbiota preparation significantly decreased FMT-associated adverse events (Zhang et al., 2020).

### 5.2 Centrifugation

Centrifugation methods include simple high-speed centrifugation, differential centrifugation, and density gradient centrifugation (Stevens and Jaykus, 2004; Hinzke et al., 2018).

#### 5.2.1 Simple high-speed centrifugation (<60,000 × g)

Most studies employ simple centrifugation, although the operational parameters vary significantly across protocols: 500 × g for 5 min (Stebegg et al., 2019), 800 × g for 3 min (Lai et al., 2018), 2000 × g for 5 min (Liu et al., 2020), 3,000 × g for 10 min (Randolph et al., 2025), 5,000 × g for 20 min (Randolph et al., 2025; Zhou et al., 2019) and 6,000 × g for 15 min (Perez et al., 2016; Hu et al., 2018).

#### 5.2.2 Differential centrifugation

Many researchers have employed simple centrifugation methods to concentrate the gut microbiota after filtration to eliminate undigested small particulates in the fecal suspension. Others have used low-speed centrifugation to remove heavier particles, followed by high-speed centrifugation to sediment bacterial cells. In general, applying centrifugation at extremely low speeds (<1,000 × g) promotes sedimentation of fecal debris, leaving bacterial cells in the supernatant; when applied at slightly higher g-forces (1,000–8,000 × g), bacterial cells, as well as other particles of equal or greater density, will sediment (Stevens and Jaykus, 2004). For example, Sedeek SA et al. spun at 400 × g to eliminate large food particles, followed by a second centrifugation at 6,000 × g to collect the gut microbiota (Sedeek et al., 2025).

#### 5.2.3 Density gradient centrifugation

Differential and density gradient centrifugation are more prevalent in microbiota purification or targeted bacterial isolation studies.

Current methodologies for preparing fecal microbiota suspensions are characterized by inconsistent reporting of centrifugation parameters, with a significant proportion of studies failing to document these operational details (Sharon et al., 2019; Wang et al., 2025). A high centrifugal force can cause a substantial decrease in the viability and pathogenicity of bacterial cells (Peterson Brandon, 2012; Sharifian and Norouzi, 2023); in contrast, the application of a low centrifugal force (or time) might lead to the loss of bacterial cells in the supernatant (Sharifian and Norouzi, 2023). Optimizing the crucial parameters in bacterial centrifugation will increase separation efficiency and increase the quality of isolated cellular components, which has significant implications for the advancement of FMT research.

## 6 Cryopreservation

The fecal suspension needs to be frozen and stored until it is used by humans or animals after laboratory preparation. Cryoprotection before freezing is crucial for preserving bacterial cell viability and integrity (Fadda, 2020).

With respect to cryopreservation, microbial viability is predominantly influenced by storage duration and temperature. Current guidelines recommend storage at −80°C for no longer than 6 months and at −20°C for a maximum of 1 month (Cammarota et al., 2017; Chen, 2022; Mullish et al., 2018). Prolonged storage at −20°C may compromise the stability of clinical outcomes in FMT, particularly concerning the abundance of Actinobacteria and Bacteroidetes (Nicco et al., 2020). For thawing, samples should be rapidly warmed in a 37°C water bath and utilized within 6 h (Cammarota et al., 2017). Current comparative analyses of thawing procedures have demonstrated that a 5-min thawing period at 37°C significantly enhances bacterial viability preservation (Bottino et al., 2024).

Zhang et al. emphasized that microbiotherapy formulation descriptions should detail microbial states (e.g., fresh, frozen, lyophilized), subsidiary materials, and storage methods. The guidelines note that microbial states may correlate with clinical outcomes, underscoring the importance of precise reporting in therapeutic applications (Tong et al., 2021). In cryoprotectants, glycerol remains the widely used and well-established agent in FMT liquid cryopreservation. Under-80°C conditions, FMT samples preserved with 10% glycerol maintain stable microbial community structures for up to 6 months (Reygner et al., 2020). However, glycerol’s limitations include its unsuitability for lyophilization. Due to its high viscosity and low vapor pressure, glycerol-containing samples tend to form sticky, moist lumps rather than dry, porous powder after freeze-drying, creating significant challenges for subsequent pulverization and capsule encapsulation (Bokoliya et al., 2021; Staley et al., 2017). In contrast, trehalose demonstrates superior protection in lyophilized FMT formulations, maintaining bacterial viability comparable to fresh samples. A study selected 5% trehalose as the standard cryoprotectant for lyophilized FMT, showing better efficacy than mannitol (Staley et al., 2017). Maltodextrin is rarely used alone as a primary cryoprotectant, but its value lies in combination with trehalose, particularly in oral capsule preparation. For instance, a 3:1 or 1:3 mixture of maltodextrin and trehalose has proven effective in preserving the viability of lyophilized FMT grafts (Bokoliya et al., 2021).

## 7 Dosage

No standardized dosing regimen exists for FMT tailored to specific disease indications. Previous studies have predominantly described FMT dosage using volumetric (mL) or weight-based (g) units, complicating cross-study comparisons due to inconsistent quantification methods.

Carlson *et al*. emphasized that FMT product manufacturing necessitates potency assays to quantify viable microbial content as part of release specifications and stability evaluations; validated methods to estimate total viable organisms may include colony-forming unit (CFU) enumeration via serial dilution plating (despite underestimating unculturable taxa), membrane-impermeable dye exclusion (e.g., live/dead differentiation by microscopy/flow cytometry), or qPCR combined with propidium monoazide pretreatment (Carlson, 2020). These assays must demonstrate product stability throughout clinical trials and enable longitudinal viability comparisons between batches (Carlson, 2020). Traditional plate counting methods are time-consuming, labor-intensive, and have low throughput, failing to culture all viable but non-culturable bacteria. To overcome these limitations, various emerging technologies have emerged, providing new solutions for precise quantification of viable bacteria in FMT. For instance, the digital colony-forming unit assay—a microfluidic-based innovation—divides diluted bacterial samples into thousands of tiny, independent droplets. Cultured bacterial droplets change color or emit fluorescence, while non-bacterial ones remain unchanged. By analyzing the proportion of positive droplets using Poisson distribution, the original bacterial concentration can be accurately calculated (Scheler et al., 2017). Meanwhile, we note that qPCR quantifies microorganisms by detecting specific gene copies (e.g., 16S rRNA). Although qPCR cannot distinguish between live and dead bacterial DNA, it can be combined with pre-treatment techniques like propidium azide bromide (PMA). PMA selectively binds to dead cells and covalently attaches to DNA, inhibiting subsequent PCR amplification. Thus, PMA-qPCR enables specific quantification of viable bacteria (Kralik et al., 2025). This method bypasses the culture step, allowing detection of viable but non-culturable bacteria and other viable bacteria, providing a more comprehensive bacterial profile than traditional CFU methods.

According to Zhang *et al*., in the PRIM Guidelines, it is crucial to detail the dose (e.g., bacterial count, colony forming unit) and frequency (e.g., treatment timeline) to increase the clarity and reproducibility of microbiotherapy studies (Zhang, 2025). These authors noted that these factors can significantly influence treatment efficacy (Zhang, 2025). For example, higher doses may increase response rates. Additionally, the colony forming unit or bacterial count should be reported when applicable (Zhang, 2025). Chen et al. conducted a dose-ranging study in DSS-induced colitis mice, administering the CLA-producing *Bifidobacterium breve* CCFM683 at daily doses spanning 10^6^ to 10^10^ CFU. They observed that only the two highest doses—10^9^ and 10^10^ CFU/day—conferred significant amelioration of colitis symptoms (Chen et al., 2023). The consensus recommends that the viable bacteria count after resuscitation meets the requirement of a basic clinical unit dose of 10^13^ CFU (Shi and Zhang, 2020), with each transplant requiring ≥2.5 × 10^12^ CFU (Chen, 2022). Dosage also varies with transplantation method. In colonoscopy-assisted transplants, the dose is 1–5 U per procedure (1 U = 1.0 × 10^13^ bacteria) once daily. In capsule-based transplants, adults need ≥30 g of fecal material, with 30 capsules containing a median of 38.6 (24.0–56.7) g of fecal microbiota (Chen, 2022).

## 8 Operating environment

To ensure bacterial abundance and microbial community structure, in addition to the selection of appropriate suspension buffers and purification methodologies, particular attention should be given to the preparation environment of fecal microbiota suspensions. Current evidence indicates that anaerobic bacteria dominate the human gut microbiota, exceeding their aerobic counterparts by more than three orders of magnitude (Finegold, 1995). Given the heightened oxygen sensitivity of anaerobes, improper environmental control during processing may induce anaerobic bacterial death (Carlson, 2020; Rigottier-Gois, 2013).

Chu et al. demonstrated that oxygen exposure during fecal homogenization significantly modifies the taxonomic composition of viable bacterial communities, with a pronounced detrimental impact on Firmicutes abundance (Chu et al., 2017). Ben-Amor et al. reported an immediate decline in viability to 49% within <2 min of atmospheric oxygen exposure (Ben-Amor et al., 2005), whereas Brusa et al. reported that 4–5 min of ambient oxygenation reduced anaerobic survival rates to ~50%, followed by catastrophic depletion (0.1% survival at 40 min; near-total elimination by 2 h; Brusa et al., 1989). This temporal gradient of oxygen damage was further quantified by Bellali et al., who reported 61.69% survival after ≤2 min of exposure, with a moderate decline to 55.52% over 90 min (Bellali et al., 2019).

Comparative analyses reveal nuanced impacts of processing conditions. Berta et al. reported that strict anaerobic handling better preserved microbial diversity than aerobic methods did, although the differences among groups were not statistically significant (Bosch et al., 2023). In contrast, Papanicolas et al. reported a 31% disparity in mortality between anaerobic preservation and oxygen-exposed samples, with normoxic conditions disproportionately eliminating the keystone taxa *Faecalibacterium prausnitzii* (butyrate producer), *Bacteroides profundus*, *Bifidobacterium adolescentis/longum* (immunomodulators), *Roseburia* spp. (fiber degrader), and *Bacteroides anaerophilus* (Papanicolas et al., 2019). These oxygen-sensitive organisms collectively regulate mucosal immunity, metabolic homeostasis, and anti-inflammatory responses. Another experiment shows that direct anaerobic processing of donor stool is superior to aerobic processing conditions for preserving the bacterial viability of obligate anaerobes and butyrate-producing bacteria related to the clinical response to FMT in ulcerative colitis patients, including *Faecalibacterium*, *Eubacterium hallii*, and *Blautia* (Bénard et al., 2023).

Overall, ≥2 min of atmospheric exposure induces ~50% microbial mortality, which escalates rapidly with prolonged oxygenation. This vulnerability profile necessitates optimized anaerobic workflows for fecal suspension preparation. Nevertheless, certain facultative anaerobes (e.g., spore-forming Clostridiales) exhibit extracellular persistence through resilient sporulation mechanisms, potentially enabling microbial transmission despite oxygen-induced community perturbations (Setlow, 2007).

To establish an anaerobic environment, anaerobic sampling bags or containers can be employed at the point of collection; a chemical oxygen-scavenging sachet (e.g., Anaeropack®) rapidly consumes residual oxygen to generate a micro- or strictly anaerobic milieu (Shimizu et al., 2021). During processing, an anaerobic workstation should be utilized, and oxygen concentration must be monitored throughout the workflow. The workstation maintains a continuous inert-gas supply, recirculating purification, and catalytic reactions to create and sustain an oxygen-free workspace.

## 9 Fecal transplantation method

Current FMT preparations mainly include bacterial suspensions (for enteroscopy, nasogastric, or nasojejunal administration; Varga et al., 2021; Li et al., 2024; Scheperjans et al., 2024) and capsules (Varga et al., 2023; Vigvári et al., 2019; Gómez-Pérez et al., 2024). The bacterial suspension, the most traditional form, is simple to prepare and can be administered via various routes, such as nasogastric or nasojejunal tubes or colonoscopy, making it suitable for rapid microbiota transplantation. However, it has a short shelf-life (under 24 h), needs strict cold-chain storage and transport (Youngster et al., 2014), and its invasive administration may be poorly tolerated by patients. In contrast, FMT capsules can be self-administered by patients without medical assistance, greatly improving their acceptance and adherence (Gweon and Na, 2021). For the preparation of oral capsules in the context of FMT, freeze drying is commonly employed. Freeze drying refers to the process in which the bacterial suspension is first frozen and then subjected to sublimation drying under vacuum to remove water, thereby forming a stable bacterial powder. During this process, lyoprotectants (e.g., trehalose, skim milk powder, etc.) are usually added to improve microbial survival during freeze drying and rehydration (Hansen et al., 2024; Sipos et al., 2024). The freeze-drying process protects bacterial viability, with freeze drying capsules preserving bacterial activity for up to 12 months at 4 °C (Reygner et al., 2020). This way also allows the bacteria to remain stable at room temperature for a certain period of time, greatly facilitating transportation and storage (Kamath et al., 2025). Additionally, capsule-based FMT is less time-consuming and less expensive than liquid suspension FMT via colonoscopy or nasojejunal tubes (Hansen et al., 2024). Retrospective studies have shown that the clinical remission rate in the FMT capsule group (79.7%) was significantly higher than that of the enema group (53.3%), demonstrating a stronger advantage in preventing recurrent Clostridioides difficile infections (Svensson et al., 2022). However, research on irritable bowel syndrome suggests that the enema route outperforms capsules in symptom improvement (Dai et al., 2024). Additionally, studies indicate that nasal jejunal route may achieve better efficacy in treating chronic transit constipation compared to capsules (Tian et al., 2020). These findings suggest that the optimal administration route for therapeutic outcomes likely depends on both the pathophysiological characteristics of the target disease and the specific intestinal regions where microbial communities need to act.

## 10 Conclusion

This review comprehensively examines the current laboratory methods used in the preparation of fecal suspensions for FMT, including sample collection, suspension buffers, homogenization, purification, filtration, centrifugation, cryopreservation, dosage, the operating environment, and the transplantation form. By analyzing the different operations and their indications at each preparation step, this review aims to provide a valuable reference for the laboratory preparation of fecal suspensions as shown in Table 1. It is undeniable that FMT still requires extensive clinical research to standardize the manufacturing processes of delivery systems such as capsules. A reproducible and traceable standard operating procedure needs to be established to encompass the entire workflow—from fecal collection and microbial isolation to storage, transport, and eventual clinical administration. Concurrently, novel quality-control strategies grounded in integrated microbiome–metabolome profiling are anticipated to enable dynamic correction of microbiota formulation quality and activity through real-time monitoring of key metabolites such as short-chain fatty acids and bile acids, thereby achieving precision quality control. In conclusion, the development of more standardized and optimized protocols for preparing fecal suspensions is essential to enhance the therapeutic potential and safety of FMT, thereby facilitating its broader application in clinical practice.

**Table 1:** Preparation steps and related precautions for laboratory fecal suspensions.

Step		Point		Attention
Sample collection		Transport at 4°C, transit time≤ 6 h Recommended sample size ≥ 50 g		Ensures sample viability and minimizes microbial changes during transport. Strict temperature and time control needed. Larger sample sizes may be logistically challenging.
Suspension buffers		PBS or normal saline Additives like cysteine hydrochloride for protection Fecal-to-buffer ratios 1:3 to 1:10 (w/v)		Maintains microbial viability and suspension quality Additives protect anaerobic bacteria.
Operating environment		Anaerobic handling		Preserves oxygen-sensitive taxa critical for therapeutic effects.
Homogenization		Manual stirring Vortex mixing Mechanical oscillation Blender		Ensures even microbial distribution
Purification	Filtration	Sterile gauze Plastic membranes Metal mesh sieves	Coarse filtration Combined with centrifugation Microfiltration combined with centrifugation	
	Centrifugation	Simple centrifugation Differential centrifugation Density gradient centrifugation	Parameters vary: 500 × g-6,000 × g, 3–20 min	Low-speed centrifugation is used for particulate removal High-speed centrifugation is used for facilitate bacterial concentration. Differential and density gradient centrifugation are more prevalent in microbiota purification or targeted bacterial isolation studies
Cryopreservation		Cryoprotectants: glycerol, dimethyl sulfoxide, maltodextrin, trehalose Storage: −80°C for ≤ 6 months, −20°C for ≤ 1 month Thawing: rapid warming in 37°C water bath		Storage duration and temperature critical Improper thawing reduces viability.
Dosage		Optical density; Colony-forming units (CFU): ≥ 10^13^ CFU per dose		
Transplantation Form		Bacterial suspensions; capsules		Bacterial suspensions: short shelf-life, invasive administration Capsules: self-administration, room-temperature stability
